# Retention of Artificial Dentures

**Published:** 1894-05

**Authors:** 


ARTICLE VI.

### RETENTION OF ARTIFICIAL DENTURES.

Dr. Storer Bennett, of England, advises the following: The aim of the experiments was to produce hinged bands, whereby teeth might be clasped around their most constricted portions, unimpeded, and even assisted by the overhanging portions of their more accurately fitting, and in many instances smaller plates can be used. The bands are of two varieties, the rest being self-adjusting, on the principle of the spring rings frequently attached to watch chains; the other a modification of the ordinary brooch joint.

To make the self-adjusting band: (1) Take a piece of thin gold tube about ⅜ inch in length. (2) Make solid at one end for about 1-10 inch by soldering into it a piece of gold wire. (3) Tap the opposite extremity with a screw for 1-32 inch. (4) Saw a slot through the middle of the solid end of the tube parallel to its long axis, extending as far back as the hollow part, and rather farther on one side than the other. (5) Fit a small piece of flat gold into the slot to form a tongue or central portion of a hinge, the width of the tube, but projecting slightly beyond its anterior extremity. (6) Drill a hole in the solid portion of the tube and tongue at right angles to the slot. (7) Pass a pin through them, the pin being ultimately rivetted or screwed. A band having been accurately fitted to the model, is soldered to the anterior extremity of the tongue, and when this is replaced in the slot and transfixed by the pin the whole forms a hinged hand. A piece of extended spiral spring is now thrust into the posterior end of the tube till it comes in contact with the back portion of the tongue, and is then held in position by a small gold plug screwed into the tube behind it.

It will greatly facilitate the accurate cuttin of this slot if a small steel tube with a slot in one end be used as a guide, and if a hole is drilled in the template across the slot it ensures the hole in the gold tube being properly placed.

The band so formed may be attached to a gold plate by removing the spring and soldering the tube in the most suitable position, the spring being subsequently replaced and retained by the screw plug.

Where vulcanite is to be brought anywhere near the huge, it is necessary before packing, so as to exclude the rubber, to introduce a little oxiphosphate, which may be subsequently dissolved with hydrochloric acid. The case is then ready for use.

It sometimes happens that it is convenient to have a band which can be opened and left in this position while the plate is being inserted or removed from the mouth; the band then being closed, and remaining firmly fixed till intentionally opened again. In this case the band is soldered to the piece of tube which forms the central portion of a brooch joint, the band being carried sufficiently beyond the tube to press on a small flat spring, bent into the shape of a horseshoe magnet. This causes the joint to open or close with a snap, the spring locking the band securely in either position. To prevent the ingress of rubber during packing, or of food when in use, the joint and spring are enclosed in a small gold box (measuring only 3-16 inch in its largest diameter), which can be soldered to a plate or embedded in vulcanite.

Sometimes one or two teeth have been lost on one side of the mouth only, the resulting space being wedge-shaped, with the base toward the gum, owing to the tilting of the adjoining teeth. Such cases are usually treated, if treated at all, by inserting a plate which covers a large portion of the mouth, to insure steadiness and safety. So large a plate is a source of much inconvenience to the wearer, and the patient is frequently dissuaded from having such a gap filled up at all. The smaller plates can be made—consistent with their safety, steadiness and ease of of insertion and removal—the better for the patient and the teeth.

By the use of the locking bands referred to, the treatment of such cases becomes easy. A plate no larger than the space, but which it accurately fits, has ordinary bands adjusted to the lingual surface of the tooth in front and behind the gap. Two locking bands are then adapted to the labial surfaces of these teeth in such a way that, when open, they are no wider than the width of the gap. Such a plate, when placed in the mouth with the bands open, may be dovetailed into position by pushing it outward from the lingual toward the buccal surface, and is secured by closing the bands, the overhanging crowns effectually preventing any upward displacement.

If the loss of a front tooth leaves such a wedge-shaped space with the base toward the gum, a plate tooth may be attached to a small plate by means of a brooch joint in such a way that the tooth may be placed in position by turning up the teeth completely out of the way. The plate may then be placed in the wedge-shaped gap, dovetailed into position, and the spring holds it firmly in its place. The method is as follows: A very small plate is used which accurately fits the gap; a thin flat tooth, having the pins as near to the neck as possible, is backed and fitted in the usual manner, so that a small band may be fitted on to the buccal surface of the necks of the adjoining teeth. The small plate has a very small band passing behind the tooth on either side of the lingual surface to prevent the plate being dislocated forward. Next a piece of 12 karat plate has two parallel lines cut into it, so as to somewhat resemble a comb with three equal teeth. On the other part of the comb a small piece of tube is soldered. This middle piece of tube is then soldered on to the backing of the tooth, a pin is run through the hole, forming a common brooch joint which allows the tooth to move upward and downward on the comb. The back of the comb is now soldered to the plate immediately behind the backing of the tooth. If now a pin is run through all the three pieces of tube so as to unite the unattached teeth to the plate itself, it gives a tooth working on a hinged joint, which may be turned up entirely while the plate is being dovetailed into position, and may then be closed down. If at the same time a little tongue of gold is soldered to the central tube so as to press on the middle portion of gum, this being a spring will press on and retain the artificial teeth either in a horizontal or vertical position. When the buccal bands are too conspicuous, a very small plate is fitted accurately and reduced to the size of the nearest part of the gap. An ordinary tooth is then taken and backed in the usual manner; two blades are then fitted, one of them soldered to the plate. On the back of the backing of the tooth a piece of small gold tube is soldered, running transversely across the gap, one end of the gap being closed by the plate and the other end open. A small slot is now cut parallel to the long axis of the tube, a piece of wire is then soldered in such a position as to be able to travel outward and inward. Now, if a small piece of open spiral spring is thrust into the tube before putting the wire in its place, the spiral spring will tend to throw the band outward toward the tooth. The objection may be made that the pressure of the spring on the natural teeth will loosen or displace them, but observation for a considerable time shows this does not happen, because of the weakness of the spring—*Dental Record.*

